# An integrated, collaborative healthcare model for the early diagnosis and management of dementia: Preliminary audit results from the first transdisciplinary service integrating family medicine and geriatric psychiatry services to the heart of patients’ homes

**DOI:** 10.1186/s12888-019-2033-7

**Published:** 2019-02-08

**Authors:** Shan Hui Lai, Tung Tsoi, Chao Tian Tang, Richard Jor Yeong Hui, Kim Kiat Tan, Yehudi Wee Shung Yeo, Ee Heok Kua

**Affiliations:** 1 Choa Chu Kang Polyclinic, National University Polyclinics, 2 Teck Whye Crescent #01-00, Singapore, 688846 Singapore; 2 Department of Psychological Medicine, National University Hospital, NUHS Tower Block Level 9, 1E, Kent Ridge Road, 119228 Singapore; 3 National Healthcare Group(NHG) National Psychiatry Residency Programme, 3 Fusionopolis Link #03-08, Nexus@one-north, Singapore, 138543 Singapore

**Keywords:** Dementia, Geriatric Psychiatry, Family Medicine, Collaboration

## Abstract

**Background:**

The number of dementia cases is expected to rise exponentially over the years in many parts of the world. Collaborative healthcare partnerships are envisaged as a solution to this problem. Primary care physicians form the vanguard of early detection of dementia and influence clinical care that these patients receive. However, evidence suggests that they will benefit from closer support from specialist services in dementia care. An interdisciplinary, collaborative memory clinic was established in 2012 as a collaborative effort between a large family medicine based service and a specialist geriatric psychiatry service in Singapore. It is the first service in the world that integrates a family medicine based service with geriatric psychiatry expertise in conjunction with community-based partnerships in an effort to provide holistic, integrated care right into the heart of patients’ homes as well as training in dementia care for family medicine physicians. We describe our model of care and the preliminary findings of our audit on the results of this new model of care.

**Methods:**

This was a retrospective audit done on the electronic medical records of all patients seen at the Memory Clinic in Choa Chu Kang Polyclinic from August 2013 to March 2016. The information collected included gender, referral source, patient trajectories, presence of behavioural and psychological symptoms of dementia and percentage of caregivers found to be in need of support. A detailed outline of the service workflow and processes were described.

**Results:**

A majority (93.5%) of the patients had their memory problems managed at the memory clinic without escalation to other specialist services. 22.7% of patients presented with behavioural and psychological symptoms of dementia. When initially assessed, a majority (82.2%) of patients’ caregivers were found to be in need of support with 99.5% of such caregivers’ needs addressed with memory clinic services.

**Conclusion:**

Our model of care has the potential to shape future dementia care in Singapore and other countries with a similar healthcare setting. Redesigning and evolving healthcare services to promote close collaboration between primary care practitioners and specialist services for dementia care can facilitate seamless delivery of care for the benefit of patients.

## Background

Collaborative healthcare partnerships are envisaged as the next frontier in healthcare with the growing need to provide expeditious yet cost effective care for an ever-increasing ageing population in many countries. In 2050, around 20% of the projected world population of 9 billion is expected to be aged over 60-years, compared to around 10% in 2000 [1]. As one of the fastest aging populations in Asia, Singapore faces the challenge of developing new strategies to accommodate this changing age structure. Currently, 7% of Singapore’s population is over the age of 65, but this is projected to increase to 19% by 2030 [2]. The incidence of dementia increases sharply with age and because of the increase in life expectancy, the number of dementia cases is expected to rise dramatically over time [3].Correspondingly, the estimated number of dementia patients in Singapore is expected to increase exponentially from 45,000 in 2015, to 103,000 in 2030 and eventually to 241,000 in 2050 [4].

General practitioners are often the first physicians contacted by patients suffering from dementia or their caregivers. Hence, they form the vanguard of early detection of dementia with considerable influence on the subsequent diagnostic process and clinical care that these patients receive [5]. In high income countries, only 20–50% of dementia cases are recognized and documented in primary care with a much greater treatment gap in low and middle income countries [6]. If extrapolated to other countries worldwide, these statistics suggest that approximately 28 million of the 36 million people with dementia have not received a diagnosis [6]. A systematic review of the literature revealed that the true prevalence of missed and delayed diagnoses of dementia is unknown but appears to be high [7]. Major contributory factors were identified as problems with provider attitudes and patient-provider communication, educational deficits, and system resource constraints [7]. In addition, the diagnosis and management of patients with dementing illnesses can uncertain as the underlying causes can present clinically in different ways that mask their true nature or may even be a manifestation of confounding comorbid conditions [8].

Primary care physicians in many different countries have reported difficulties with the diagnosis and management of dementia. In the United Kingdom, a survey of general practitioners revealed that one third of general practitioners sampled expressed limited confidence in their diagnostic skills, whilst two-thirds lacked confidence in management of behaviour and other problems in dementia [9]. The main difficulties identified by the participants were talking with patients about the diagnosis, responding to behaviour problems and coordinating support services [9]. Another qualitative study done to study general practitioner attitudes and practices in the United Kingdom revealed that there was indecision regarding the efficacy of available medications, uncertainty of the diagnostic process with uncertainty in their ability to follow the correct procedures to diagnose dementia [10]. In a large survey of European and Mediterranean countries, primary care physicians who were surveyed with regards to diagnosing dementia on their own showed that majority participants in six countries including Finland, Hungary, Malta, Poland, Romania and Turkey rarely or never tried to establish a diagnosis of dementia on their own [11]. In Kathmandu, Nepal, a study to identify general practitioners’ knowledge, practices, and obstacles with regard to the diagnosis and management of dementia revealed that the knowledge of practitioners was unsatisfactory with nearly half of the respondents rating themselves as either a little confident or not confident at all with the diagnosis and management of dementia [12]. A recent survey on general practitioner attitudes and confidence in managing patients with dementia in Singapore revealed that while almost all general practitioners thought that the early diagnosis and management of dementia was important, only half agreed that they felt confident about diagnosing dementia [13]. Clearly, there is evidence that difficulties with the diagnosis and management of dementia in primary care pervade across continents and healthcare systems.

Hence, to an extent, one bottleneck of dementia care lies in the diagnosis. Without formal diagnosis of dementia, patients and their caregivers are not able to access the support and service that they desperately need. However, specialists in tertiary institutions mainly do the diagnosis of dementia in Singapore. Being the usual first contact point for patients and the foundation of Singapore’s health care system, family physicians can assume an important role in the early diagnosis and subsequent management of dementia cases. Memory clinics were first described in the US in the 1980’s with their purpose being the identification, investigation and treatment of memory disorders including dementia [14]. Memory clinics generally attempt to provide a multidisciplinary approach to the diagnosis and treatment of memory impairment and dementia [15]. Memory clinics run by single specialties have been set up globally in recent years in a response towards the rising tide of dementia with increased training efforts as well. One such example is in Canada where a 5 day inter-professional training program for primary care practitioners to set up memory clinics had been conducted to increase capacity for dementia care with the expectation for the participants to set up independent memory clinics [16]. Some services function to enhance the care that family physicians can provide at a primary care level but does not aim to replace the role of the patient’s own family physician or the role of the consultant, who remains an invaluable resource for more challenging concerns [17]. While there is a large focus on patients in many of these services, in many Asian countries however, families assume an important role in taking care of and supporting their older relatives. Family caregiver burden is a multidimensional phenomenon, involving their mental health, physical condition, social life, financial status and functioning of the family as a whole. It is not limited to any particular society or culture [18]. Thus, the government has the responsibility of establishing policies and services to support these family caregivers. [18]

In the primary care setting in Singapore, there are 2 tiers of expertise. The first level consists of a fully qualified doctor who has completed his/her basic medical degree without additional training in any particular areas. The 2nd level of care consists of a family medicine specialist who has undergone additional training in the field of family medicine, who would possess additional qualifications such as a master’s in family medicine. They would usually be clinical leaders in this field and provide care for more complex patients while supervising trainees in this field.

The interdisciplinary, collaborative memory clinic was established in 2012 as a collaborative effort between the Choa Chu Kang polyclinic, a large primary care facility located in the North-West of Singapore and the geriatric psychiatry service of the National University Hospital, Singapore which was a first in Singapore. Its dual purpose was to train family medicine physicians to be competent in the diagnosis and management of uncomplicated dementia cases at the primary care level but at the same time to provide a seamless pathway integrating home based services with key community partners to provide home based care services to meet the needs of patients and their families. A multidisciplinary team consisting of family medicine physicians who have received intensive training by a specialist psycho-geriatrician who also provides close support in challenging cases, allied health professionals such as case managers and social workers in conjunction with key community partners manage the clinic.

To our knowledge, this is the first service in the world, which integrates a family medicine based service with geriatric psychiatry expertise in conjunction with community-based partnerships in an effort to provide holistic, integrated care right into the heart of patients' homes.

## Methods

This was a retrospective audit done on the electronic medical records of all patients seen at the memory clinic from August 2013 to March 2016. The information collected included gender, referral source, patient trajectories, presence of behavioural and psychological symptoms of dementia and percentage of caregivers found to be in need of support. Ethics approval was not required as exemption was obtained from the National Healthcare Group’s Domain Specific Review Board (DSRB) (reference number 2016/01054).

### Patient selection

The inclusion criteria for a referral to this service was that of patients aged 65 years and above with uncomplicated complaints of memory loss for 6 months or more. Patients who needed specialist assessment for legal reasons or mental capacity certification were excluded. Prior to being seen, these patients would have basic blood investigations done including a full blood count, vitamin B12, a liver function test, thyroid function test, electrolytes such as calcium, sodium, potassium and creatinine, an electrocardiogram(ECG) and when indicated, a computed tomography (CT) scan of brain for selected cases. Caregivers would be required to attend the consultation with their caregivers for corroborative history as well.

### Workflow

The clinic accepted referrals from other primary care doctors or other sources for patients aged 65 years and above with uncomplicated complaints of memory loss for 6 months or more which fulfilled the inclusion criteria.

### Pre-appointment

Prior to the appointment, blood investigations including a full blood count, vitamin B12, a liver function test, thyroid function test, electrolytes such as calcium, sodium, potassium and creatinine and an electrocardiogram(ECG) will be ordered by the referring doctors. Caregivers were reminded to attend the consultation for corroborative history.

### First memory clinic consultation

The family physician running the memory clinic is involved assessing the initial referral, and review of all followed up cases. The core members of the team are: family physician, psycho-geriatrician, case manager, and community partners. Only the community partners do home visits. The information gathered from the home visits are conveyed via written information in the form of memos and home assessment checklists to the family physician via correspondence which is recorded electronically.

A case manager would first perform a Mini Mental State Examination (MMSE) for the patient and conduct a Zarit Burden Interview with the caregiver to assess for any caregiver stress as part of the initial assessment prior to any diagnosis. A functional assessment will also be done when necessary to identify patients’ functional status at home and the community.

After seeing the case manager, the patient will be attended to by both the family physician and the consultant psycho-geriatrician, who will be sitting side by side doing a joint-consultation. A diagnosis is made, and any behavioural, psychological and social problems of dementia, or care giver stress will be identified. A care plan, which includes both pharmacological and non-pharmacological interventions, will be formulated.

If indicated, a referral to the social worker or the community partners will be arranged for the introduction and matching of the patient and their caregivers to the relevant support schemes and agencies.

The consults and referrals are usually arranged consecutively on the same day, in adjacent rooms, but not simultaneously in a group.

### Post assessment home visits

Home visits may be conducted by these community partners to better understand the needs of these patients and their care-givers. During home visits, the patients were be assessed according to a Home Safety Checklist developed by the team, covering assessment risk factors that were difficult to evaluate in an outpatient setting. Our community partners would then monitor our patients in the community and would refer patients back to the memory clinic should the need arise. The composition of the home visit team varies depending on the projected requirements. It is usually done by a case manager with a background in nursing but with additional expertise in case management. However, prior to the home visit, if the case manager determines that a doctor is required for the case in view of ongoing complexities, the home visits will be jointly conducted by the doctor and the case manager.

### Subsequent memory clinic consultations

Patients will be reviewed in the memory clinic jointly by both the family physician and psycho-geriatrician about 2–3 months after the initial consult. The effectiveness of the various interventions implemented will be reviewed and revised when needed. Patient satisfaction surveys were routinely conducted to solicit feedback on potential avenues of improvements.

### Discharge planning from CCK memory clinic

Once these patients were stable and tolerating the medications well, they would be discharged from the memory clinic. There were several avenues for follow-up. Firstly, this included family medicine physician clinics for patients who had complex medical problems that would benefit from the care of these experienced family medicine physicians. The second option was to allow these patients to follow up with a team based care program, where a group of patients whose chronic conditions are followed up with a fixed team of doctors and case managers, for continuation of care. Patients who were very well and need minimum medical supervision were discharged to a general pool of doctors for follow-up. At any one point, should the patients become unwell again, such as the development of severe BPSD despite despite both nonpharmacological and pharmacological measures, specialist services would be enlisted for further intervention with possible admission for inpatient care if the need arose. For patients who required specialist care in a home setting, the Geriatric Psychiatry Out-Reach Assessment Consultation and Enablement (G-RACE) service at the National University Hospital which is a psychogeriatric specialist outreach service providing assessment and treatment for elderly patients with mental health conditions can be requested to follow up on these patients. This is a holistic yet dynamic system, where the patients’ level of care are matched accordingly to their needs at various points in their lives, ranging from community based support, to various clinics within the polyclinic itself to the specialist care in the hospitals. Streamlined processes with only the relevant forms were set up, to facilitate such referrals to ensure that no information is lost.

## Results

With reference to Tables 1, 489 patients were seen from August 2013 to March 2016. Among these, 266 (54.4%) were first visits and the rest were review visits. There were more female patients (66.2%) compared to male patients. The majority of patients (89.5%) were referred from within the same polyclinic while the rest were mainly referred from the National University Hospital.

**Table 1:** Profile of Memory Clinic patients managed at Choa Chu Kang polyclinic, August 2013 – March 2016

		Number of patients	Percentage
Visit type	First visit	266	54.4%
	Review visit	223	45.6%
	Total number of visits	489	100%
Gender	Male	90	33.8%
	Female	176	66.2%
	Total number of patients	266	100%
Referral Source	Other Drs in Choa Chu Kang polyclinic	239	89.5%
	Hospital specialist clinic	28	10.5%
	Total number of referrals	267	100%
Patient Trajectory	Managed in primary care memory clinic	457	93.5%
	Referred to specialist clinic	32	6.5%
	Total number of consults done	489	100%
Referred to the emergency department	Yes	8	1.6%
	No	481	98.4%
	Total number of consults	489	100%
Patients with unplanned dementia related hospital admissions	Yes	5	1%
	No	484	99%
	Total number of consults	489	100%
Behavioural and psychological symptoms of dementia	Present	111	22.7%
	Absent	378	77.3%
	Total number of consults done	489	100%
Caregivers needing support	Caregivers needing support prior to intervention	402	82.2%
	Caregivers who do not need any support	87	17.8%
	Total number consults assessed	489	100%
Among those caregivers who needs support	Caregiver needs are addressed after intervention	400	99.5%
	Care givers needs not addressed after intervention	2	0.5%
	Total number of caregiver who needs support	402	100%

Most (93.5%) of the patients had their memory problems managed at the memory clinic, with only 6.5% needed to be referred to specialists for further management. 8 patients (1.6%) were identified as having non-dementia emergent conditions and were referred to the emergency department, while 5 (1.0%) had unplanned dementia related hospital admissions. Among the 489 patients, 111 patients (22.7%) presented with psychological and behavioural symptoms of dementia. When initially assessed, 402 (82.2%) patients’ caregivers were found in need of support. 99.5% of such caregivers’ needs had been addressed by the memory clinic.

## Discussion

New models of dementia care are pivotal to ensuring timely and convenient access for assessment and care for many of these patients who would otherwise have to wait for a specialist assessment at tertiary centres. Our results have shown that having a collaborative healthcare model between primary care services and tertiary care expertise can allow for management of uncomplicated dementia cases in a primary care setting with the meeting of caregiver needs. There is an urgent need to expand the capacity of Singapore’s healthcare system in managing dementia patients, to keep pace with the rapidly aging population. Building more specialist clinics and training more specialists may not be the only solution. Our unique model with its emphasis on providing services that delivers care beyond the clinic setting to the heart of patients' homes through partnerships with community partners and home visit services represents a major move towards reducing the burden on tertiary clinical care services in this region.

A large literature review has shown that discharge planning and transitional care for patients with dementia are not adequate and are likely to lead to readmission and other poor health outcomes [19]. Many gaps exist along the trajectory of patients with dementia which may adversely affect the outcome and care of these patients in the long term ranging from the recognition of these conditions to the long term service provisions required by these patients [20, 21]. Other countries such as Canada have faced difficulties relating to the increase reliance on home care and residential care services by persons with dementia which raises critical questions about ensuring that an adequate range of services is available in local communities to support aging in place [22]. Our care model attempts to bring together the various stakeholders in the care of patients with dementia under a single coordinating unit to prevent service gaps, which can occur in many dementia services across the globe.

It is interesting to note that 93.5% of patients were managed at the memory clinic level without a referral to specialist services although 22.7% of the total patients had behavioural and psychological symptoms of dementia. This indicates that, to a certain extent, a degree of expertise and confidence by the primary care physicians in managing patients within our service in collaboration with the specialist psycho-geriatrician. While our service was not aimed at dealing with complex cases of dementia, behaviour and psychological symptoms of dementia (BPSD) would be expected in a majority of cases when observed longitudinally based on existing literature [23] [24–29]. These rates vary from 50.1% to all patients experiencing BPSD based on existing prevalence studies [27, 29]. However, a study has found that despite the fact that primary care practitioners have a wealth of knowledge about BPSD, they are largely critical of their knowledge and management skills of these symptoms, the majority reporting difficulty in diagnosing, managing as well as discriminating BPSD from other behavioural disturbances [30]. The educational aspects of our model provide opportunities for family medicine physicians to become agile and knowledgeable in the diagnosis and management of dementia as a senior psycho-geriatrician trains them.

It has been recognized that clinical practice guidelines provide evidence-based recommendations for diagnosing and managing dementia but a number of barriers prevent their implementation in the primary care setting [31]. These barriers include time constraints, the nature of dementing illness which makes it's symptoms difficult to recognize and respond to and other constraints in the primary care setting including physician attitudes [31]. Another study has shown that among providers, a major barrier often noted was the attitude that diagnosis, particularly in the early stages of dementia, was more harmful than helpful which was linked with the tendency to diagnose only when an unavoidable problem had arisen [7].

Hence, there have been calls for new models of education to provide much needed guidance and experience towards the education of primary care providers. In the United States, the model of distance education for primary care providers through biweekly multi-point videoconference virtual clinics involving local experts in the various specialties relating to dementia due to the scarcity of expert dementia care in New Mexico has proven to be an effective tool for dementia care education and training [32]. A recent systemic review on effective dementia education and training for the health and social care workforce revealed common features of the educational programs that have been found to be more efficacious [33]. This included among others the need for educational programs to be relevant to participants’ role and experience, involve active face-to-face participation, be delivered by an experienced facilitator, support application of learning in practice and underpin practice-based learning with theory [33]. Our model of care is able to incorporate all these aspects into the education of primary care providers in the diagnosis and management of dementia. This model can potentially be replicated in other polyclinics across the country and region to provide a model for post-graduate, practical continuing education in relation to dementia care.

73% of caregivers were found to be in need of support prior to intervention and the memory clinic had been able to address 81.8% of caregiver needs. Globally, at present the majority of dementia patients are living at home and cared for by a family member [34]. Informal care will be increasingly important as the number of people with dementia will rise to 65.7 million by 2030 and 115.4 million by 2050 together with a decrease of the working population [34]. Evidence suggests that individually developed multicomponent interventions including a diversity of services will decrease burden, improve quality of life, and enable caregivers to provide at-home care for longer periods prior to institutionalization [35]. Furthermore, one of the main cost driver in dementia is the cost of care provided in nursing homes whereas investing in early effective caregiver interventions may save costs in the long-term, particularly when institutionalization can be postponed [36]. Patients with dementia often require formal long-term care services because of the absence, exhaustion, or inability of family members to provide care [37]. Our findings suggest that our model of care can potentially be a way to detect caregiver needs early on at the earlier stages of dementia to avoid or minimize caregiver stress by intervening in key areas of home-based dementia care. Our close partnership with community partners to bring the focus of care to the heart of patients' homes beyond the confines of a consultation room has allowed us to provide close support to both patients and caregivers in a collaborative, yet directed manner to ensure that interventions are implemented effectively.

A systemic review to ascertain the prevalence and contributing factors for missed and delayed dementia diagnoses in primary care showed that the sensitivity of providers’ diagnoses appeared to be strongly related to dementia severity. Diagnostic accuracy was poorest among patients deemed to have few or mild symptoms of dementia. For these patients, primary care providers’ diagnostic sensitivity ranged from 0.09 to 0.41. In contrast, among patients only with severe dementia, diagnostic sensitivity ranged from to 0.60 to 1.0 [7]. This is on the background of longitudinal data from the National Alzheimer’s Coordination Center’s (NACC) research database in the United States reporting that the sensitivity of current clinical diagnostic criteria for Alzheimer’s dementia ranged from 70.9 to 87.3% with specificity ranging from 44.3 to 70.8%. This suggests that there may be substantial rates of dementia misdiagnosis among patients with cognitive impairment [38]. This is unfortunate as the treatment pathways for the various dementia pathologies differ greatly. The three most commonly used acetylcholinesterase inhibitors in Alzheimer’s dementia which are donepezil, rivastigmine, and galantamine have been studied in frontotemporal dementia and related disorders with the results so far disappointing across the board [39]. Rivastigmine, a choice for some due to its availability in patch form has also proven to have no benefit on cognition for patients with vascular dementia [40, 41]. At the same time, previous studies have shown that early diagnosis and treatment for AD can result in substantial cost savings and potentially delay institutionalization among these patients [42] [43, 44]. The significant and potentially avoidable medical resource use and related costs suggest that a more accurate diagnosis for patients with dementia could potentially be an avenue to reduce rising costs related to the care of patients with dementia. The importance of an early diagnosis of dementia cannot be understated, with a wealth of benefits exists ranging from cost effective drugs to non-drug interventions such as cognitive stimulation therapy exist that help to delay cognitive deterioration and improve quality of life [45]. Hence, the introduction of a model of care such as ours for an accurate, early detection of dementia could be one of the first steps towards evolving the diagnosis, evaluation and management of patients with dementia, taking into account limitations in specialist services and the need to grow the role of primary care physicians in the management of dementia.

The unique points of this memory clinic include a clear referral process with the necessary investigations being carried out prior to the first consultation. This has allowed for screening of reversible causes of dementia such as vitamin B12 deficiencies, hypothyroidism and electrolyte abnormalities to be diagnosed earlier. The service also houses case managers, medical social workers, and community partners all within in the same polyclinic for one-stop care. Evaluation, diagnosis and treatment of memory disorders can take significant clinician time and on-going follow-up [46]. Hence, the use of other team members can offset physician time whilst facilitate meeting the patient and caregiver needs more effectively in a timely fashion [46]. As patients with dementia often have comorbid illnesses, these patients were managed in a holistic manner in the same polyclinic instead of different providers managing their individual conditions. This integrated care of all the bio-psycho-social aspects allows us to address the needs of the patients simultaneously. Studies have shown that the diagnosis and management of co-morbid conditions such as diabetes mellitus [47] becomes poorer as dementia dominates clinical encounters and shifts attention away from the co-morbidity which can lead to increased morbidity and mortality [48, 49]. As a result, it becomes easy and convenient for the patients and caregivers to attend a single clinic and keep to their follow-up appointments. Studies have also shown that both specialist and primary care practitioners mutually agree that certain complex management issues such as complex BPSD symptoms and driving competency would require specialist input [50]. Close collaboration with specialists in our model means that the family physicians may get in touch with the specialists as soon as possible, should any uncertainties arise in the course of the patient management. This support is paramount to ensure that all patients are managed safely within the abilities of the family physicians while freeing up specialist services to dedicate additional time to more complicated cases that require more expertise and attention.

There are a number of limitations to our study. Our study did not look specifically at the types of dementia managed or the stages of dementia the patients had. The caregivers were not randomly selected to complete the satisfaction survey, hence there may have been a selection bias in that only those who felt that their needs were met completed the survey. Given that the majority of the referrals originated from other primary care physicians in the same center, should this model be replicated in other centers or countries, prior effort to promote the services of this model of care may have to be done especially to other primary care physicians or referral sources that serve the targeted population. In countries where community partners’ services are not as well developed, this model of care may need to be modified to suit the environment and culture of the region it is housed.

Despite its limitations, we believe this model of care has the potential to shape future dementia care in Singapore and other countries with a similar healthcare setting. Additional educational interventions that encompass a wider range of dementia care services as well as the more challenging aspects of BPSD management can improve the expertise of primary care physicians. Redesigning healthcare services to promote close collaboration between primary care practitioners and specialist services for dementia care can facilitate seamless delivery of care for the benefit of patients and their caregivers.
